# Aggregated *α*-synuclein and complex I deficiency: exploration of their relationship in differentiated neurons

**DOI:** 10.1038/cddis.2015.166

**Published:** 2015-07-16

**Authors:** A K Reeve, M HR Ludtmann, P R Angelova, E M Simcox, M H Horrocks, D Klenerman, S Gandhi, D M Turnbull, A Y Abramov

**Affiliations:** 1 Wellcome Trust Centre for Mitochondrial Research, Institute for Neuroscience, Newcastle University Institute for Ageing, Newcastle University, Newcastle upon Tyne, UK; 2 Department of Molecular Neuroscience, Institute of Neurology, University College London, London, UK; 3 Department of Chemistry, University of Cambridge, Lensfield Road, Cambridge, UK

## Abstract

*α*-Synuclein becomes misfolded and aggregated upon damage by various factors, for example, by reactive oxygen species. These aggregated forms have been proposed to have differential toxicities and their interaction with mitochondria may cause dysfunction within this organelle that contributes to the pathogenesis of Parkinson's disease (PD). In particular, the association of *α*-synuclein with mitochondria occurs through interaction with mitochondrial complex I and importantly defects of this protein have been linked to the pathogenesis of PD. Therefore, we investigated the relationship between aggregated *α*-synuclein and mitochondrial dysfunction, and the consequences of this interaction on cell survival. To do this, we studied the effects of *α*-synuclein on cybrid cell lines harbouring mutations in either mitochondrial complex I or IV. We found that aggregated *α*-synuclein inhibited mitochondrial complex I in control and complex IV-deficient cells. However, when aggregated *α*-synuclein was applied to complex I-deficient cells, there was no additional inhibition of mitochondrial function or increase in cell death. This would suggest that as complex I-deficient cells have already adapted to their mitochondrial defect, the subsequent toxic effects of *α*-synuclein are reduced.

The pathological hallmark of Parkinson's disease (PD) is the presence of *α*-synuclein aggregates, particularly within the substantia nigra (SN). These aggregations take the form of intracellular Lewy bodies, and also neuritic aggregations. However, both the effect of these inclusions on neuronal survival and the toxicity of different forms of *α*-synuclein are still debated. To aggregate *α*-synuclein must undergo a conformational change, however, the mechanism behind this change and subsequent aggregation in PD remains to be determined.

Mutations within the *α*-synuclein gene (SNCA (MIM 163890)) were the first to be associated with autosomal dominant PD, while more recently genome-wide association studies have suggested that single-nucleotide polymorphisms in this gene are important for sporadic PD. A widely expressed protein *α*-synuclein is important for synaptic vesicle recycling and the modulation of dopamine transmission within SN neurons.^1, 2, 3, 4, 5, 6, 7, 8^ It interacts with curved cellular membranes including those of mitochondria suggesting a possible mode of its toxicity,^9, 10, 11^ and can be imported into mitochondria in an energy-dependent manner.^9^ The accumulation of *α*-synuclein within mitochondria leads to complex I impairment, decreased mitochondrial membrane potential (Δ*Ψ*_m_) and increased reactive oxygen species (ROS) production. The occurrence of these changes is also dependent on calcium homoeostasis.^9, 12, 13^

Mitochondrial dysfunction has also been heavily implicated in the pathogenesis of PD. Early studies showed a decrease in mitochondrial complex I in the SN of PD patients and studies involving the inhibition of this complex replicate many of the features of this disease. In addition, SN neurons show high levels of mitochondrial DNA deletions in old age,^14, 15^ which lead to respiratory deficiency, and the environment of the SN is believed to be particularly oxidative due to a number of processes, including the metabolism of dopamine. More recently a number of genes known to cause autosomal recessive forms of PD have been shown to encode proteins with functions associated with mitochondrial turnover (Parkin/Pink1 (MIM 602544, MIM 608309)) or oxidative stress (DJ-1 (MIM 602533)). However, the link between these two processes and the loss of dopaminergic neurons in PD remains to be elucidated.

Several hypotheses have been suggested for what might cause *α*-synuclein to undergo the conformational change into more aggregate prone forms, from oxidative stress to gene mutations. Furthermore, the accumulation of mitochondrial DNA (mtDNA) mutations and dysfunctional mitochondria with advancing age are likely to have an effect on oxidative stress levels within the SN, which might contribute further to the misfolding and accumulation of this protein. Numerous studies have used rotenone and other toxins to induce mitochondrial dysfunction and monitor the accumulation of *α*-synuclein, despite the wealth of information that these studies provide they often do not reflect the subtleties of the slow accumulation of mitochondrial dysfunction within ageing SN neurons.

Therefore, we investigated the relationship between mitochondria and aggregated *α*-synuclein, focussing on how these forms affect neurons with and without mitochondrial dysfunction. We wanted to understand how aggregated *α*-synuclein impacted on the survival of cells with mitochondrial dysfunction, to enable a deeper understanding of the effect of these two processes on neuronal survival. To investigate this we used cells with mutations in and partial inhibition of complexes I and IV.

## Results

### Aggregated *α*-synuclein depolarises mitochondria

#### Changes in embryonic stem cell cybrids

**Figure 1:**
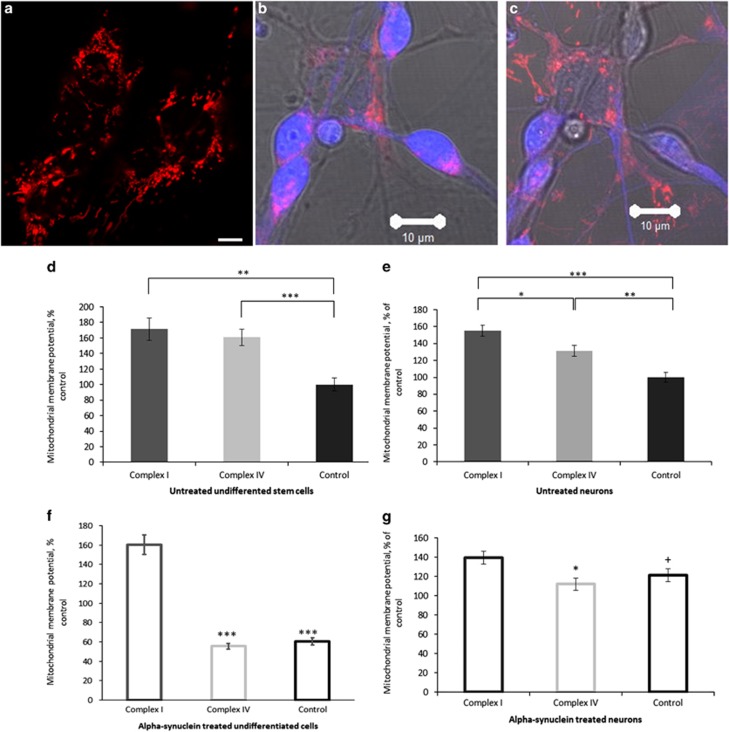
*α*-Synuclein affects mitochondrial membrane potential. TMRM fluorescence was used to quantify mitochondrial membrane potential in undifferentiated stem cells (**a**) and neurons (**b**) from complex I- and complex IV-deficient cybrids and in control cell lines. (**b**) When identifying neurons a brightfield image is used to allow the morphology of the neurons to be assessed, (**c**) Z-Stacked images show that the neurons lie in a different focal plane to non-neuronal cells. Mitochondria are only analysed from neurons. Basal TMRM fluorescence was measured (**d**) showing increased mitochondrial membrane potential in the complex I (**P=0.0004) and complex IV (***P<0.0001) stem cells compared with controls. (**f**) Membrane potential was measured again following 24 h treatment with aggregated *α*-synuclein. *α*-Synuclein significantly reduced the membrane potential of complex IV-deficient cybrids and control stem cells (***P<0.0001) but had no effect on the membrane potential of complex I-deficient cybrid stem cells. Basal TMRM fluorescence was measured in neurons (**e**) and showed increased mitochondrial membrane potential in the complex I-deficient neurons compared with the controls (***P<0.0001) and complex IV-deficient neurons (*P=0.0154) and between the complex IV-deficient neurons and the control cells (**P=0.001). (**g**) Membrane potential was measured again following 24 h treatment with aggregated *α*-synuclein. *α-S*ynuclein did not affect complex I-deficient neurons but reduced the membrane potential of complex IV-deficient neurons (^+^*P*=0.0490) and increased that of the control neurons (*P= 0.0186). Scale bar represents 10 *μ*m

Mitochondrial membrane potential (Δ*ψ*_m_) is an indicator of mitochondrial function and health. Any changes in Δ*ψ*_m_ can modify cell physiology and can be a trigger for pathology. We measured the effect of aggregated *α*-synuclein on the Δ*ψ*_m_ of both neurons and undifferentiated stem cells (Figures 1a and b). Analysis of basal TMRM fluorescence in untreated cells shows, consistent with previous data,^16^ that complex I-deficient cybrid cells show an increased membrane potential compared with controls (Figure 1c; *n*=18 (taken across three images), *P*=0.0004), whereas the Δ*ψ*_m_ of complex IV-deficient cybrid cells is also greater than controls (*n*= 18, *P*<0.0001). The basal membrane potential of complex I-deficient cybrid cells is, however, not significantly different to that of the complex IV-deficient cybrid cells (*n*= 18, *P*=0.6017).

We applied a preparation of aggregated *α*-synuclein based on the aggregation of fluorescently labelled *α*-synuclein monomers, 50% labelled with Alexa Fluor 488 (FRET (fluorescence resonance energy transfer) donor) and 50% with Alexa Fluor 647 or Alexa Fluor 594 (FRET acceptors). As oligomers form, they are statistically likely to contain both a donor and acceptor label, and FRET is able to occur between these allowing them to be separated from the vast majority of monomeric protein making up the sample. We have previously used this method to characterise *in vitro* aggregation of labelled *α*-synuclein.^17^ This study demonstrated that globular non-toxic oligomers are first formed which then structurally convert to toxic, *β*-sheet containing oligomers, and ultimately fibrils. For this study described, we halted aggregation at 29 h, and single-molecule confocal FRET analysis confirms the presence of ~0.8% oligomers (number of detected oligomers as a fraction of total number of detected events). This preparation of aggregated *α*-synuclein (mixture of 99% monomeric and ~1% oligomeric *α*-synuclein) was used for all the following experiments (with variation in the AF-label used depending on the imaging experiment). The effects of monomeric *α*-synuclein alone were tested to distinguish it from the action of the aggregated (oligomeric) peptide.

Following 24 h treatment with aggregated *α*-synuclein (100 nM), there is a significant reduction in Δ*ψ*_m_ of control and complex IV-deficient cybrid cells (*n*=18, *P*<0.0001 from 160–55% (of control) for complex IV-deficient cybrid cells and from 100–60% for controls; Figure 1e) when compared with their untreated counterparts, whereas Δ*ψ*_m_ in the complex I-deficient cybrid cells is not affected (*n*=18, *P*=0.5270).

#### Changes in differentiated neurons

Complex I-deficient neurons showed a significant increase in Δ*ψ*_m_ compared with controls (*n*=50, *P*<0.0001) and complex IV-deficient neurons (*n*=50, *P*=0.0154; Figure 1d). Complex IV-deficient neurons also show an increased Δ*ψ*_m_ compared with controls (*n*=40, *P*=0.0010).

Twenty-four hours treatment with aggregated *α*-synuclein (100 nM) produced a significant reduction in the mitochondrial membrane potential of complex IV-deficient neurons (*n*=40, *P*=0.0490, 131–112% (of control)), whereas the same treatment caused significant increase in Δ*ψ*_m_ in control neurons (*n*=50, *P*=0.0186, 100–121% Figure 1f), when compared with their untreated counterparts. Again, no change in Δ*ψ*_m_ was detected following the treatment of complex I-deficient neurons (*n*=50, *P*=0.0885).

### Aggregated *α*-synuclein changes nicotinamide adenine nucleotide (NADH) redox index

The redox state of mitochondrial NADH is a function of respiratory chain activity and substrate turnover. We measured the resting level of NADH autofluorescence in differentiated neurons, which was then expressed as the ‘redox index', a ratio of the maximally oxidised and maximally reduced signals.^18^ The dynamic range of these signals was defined by obtaining the maximally oxidised signal following treatment with 1 *μ*M carbonyl cyanide p-trifluoromethoxy-phenylhydrazone (FCCP; which stimulates maximal respiration). Under these conditions, mitochondrial NADH is fully oxidised and therefore defined as 0% reduced. The maximally reduced signal was then defined as the response to 1 mM NaCN (which fully inhibits respiration preventing NADH oxidation), which promotes maximal mitochondrial NADH reduction. Hence, under these conditions NADH is taken as being 100% reduced.

**Figure 2:**
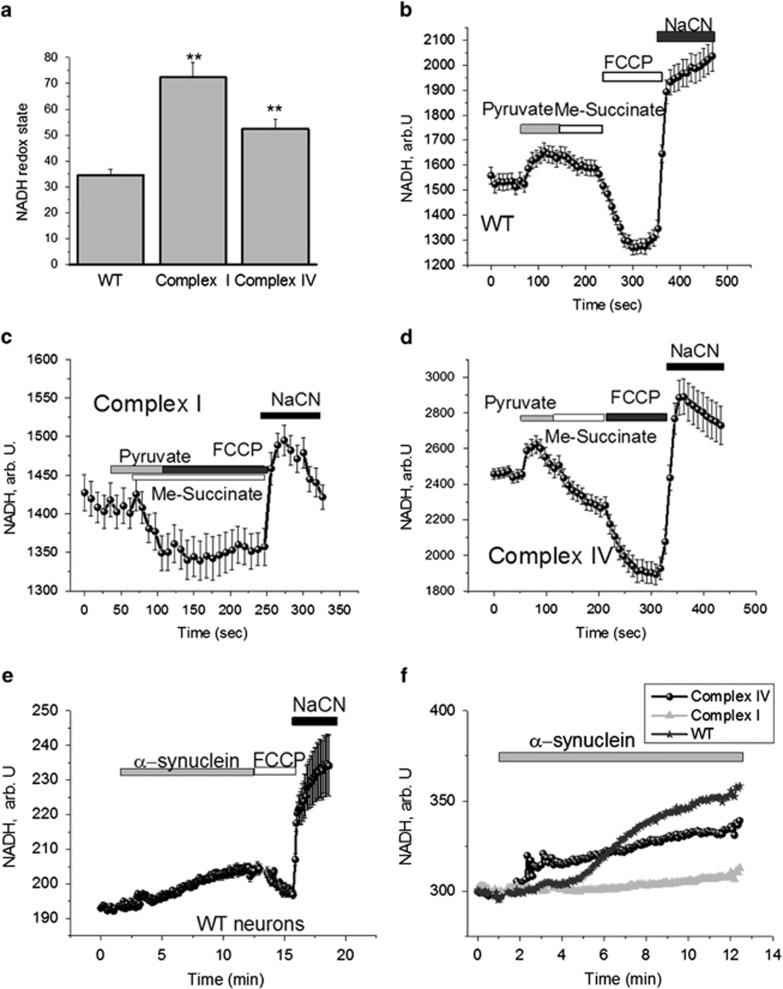
Effect of *α*-synuclein on NADH redox state in cells with mitochondrial mutations and control neurons. Estimation of the percentage change in mitochondrial NADH pool in control cells—control (**b**), complex I deficient (**c**) and cells with a deficiency of complex IV (**d**). (**a**) Redox state was estimated as: 0 is response to FCCP (maximal rate of respiration and lowest level of mitochondrial NADH) and 100% is response to cyanide (inhibition of respiration with no consumption of NADH in mitochondria); complex I- and complex IV-deficient neurons have higher NADH redox state compared with control neurons (**P<0.001). Treatment with 100 nM *α*-synuclein increased both the level of NADH autofluorescence within WT neurons and the redox state (**e**). (**f**) How the effect of *α*-synuclein treatment on WT neurons compares to neurons with mitochondrial deficiencies. An increase in NADH autofluorescence following treatment is recorded in complex IV-deficient neurons suggesting CI inhibition, but not in complex I-deficient neurons.**P<0.001

The basal redox level in complex I-deficient neurons (72.3±5.7; *n*=47; *P*<0.001) and complex IV-deficient neurons (52.4±3.8% *n*=76; *P*<0.001; Figure 2a) were significantly higher when compared with control neurons (34.6±2.2, *n*=48). For investigation of the functional activity of complex I, we used mitochondrial substrates and found that 5 mM pyruvate increased the NADH level in control and complex IV-deficient neurons but not in complex I-deficient neurons (Figures 2b and d). Interestingly, me-succinate caused oxidation of NADH in complex I- and IV-deficient neurons (Figures 2c and d), but had no effect in control neurons (Figure 2b). Thus, the increased NADH redox state in neurons with complex I and IV mutation confirms inhibition of respiration in these cells.

Application of aggregated *α*-synuclein (100 nM) induced an increase in NADH autofluorescence in both control neurons (by 31.6±2.6%, *n*=39) and cells with a complex IV deficiency (by 28.3±2.9%, *n*=33), suggesting inhibition of respiration in these cells but not in complex I-deficient cells (*n*=41; Figures 2e and f). *α*-Synuclein is known to be inhibitor of complex I.^9, 13^ We can suggest, therefore, that the absence of the effects of α-synuclein on NADH consumption in complex I-deficient cells is because this complex is already inhibited in mitochondria of these cells, because of the two mtDNA mutations within these cells.

### Effect of aggregated *α*-synuclein on cellular respiration

**Figure 3:**
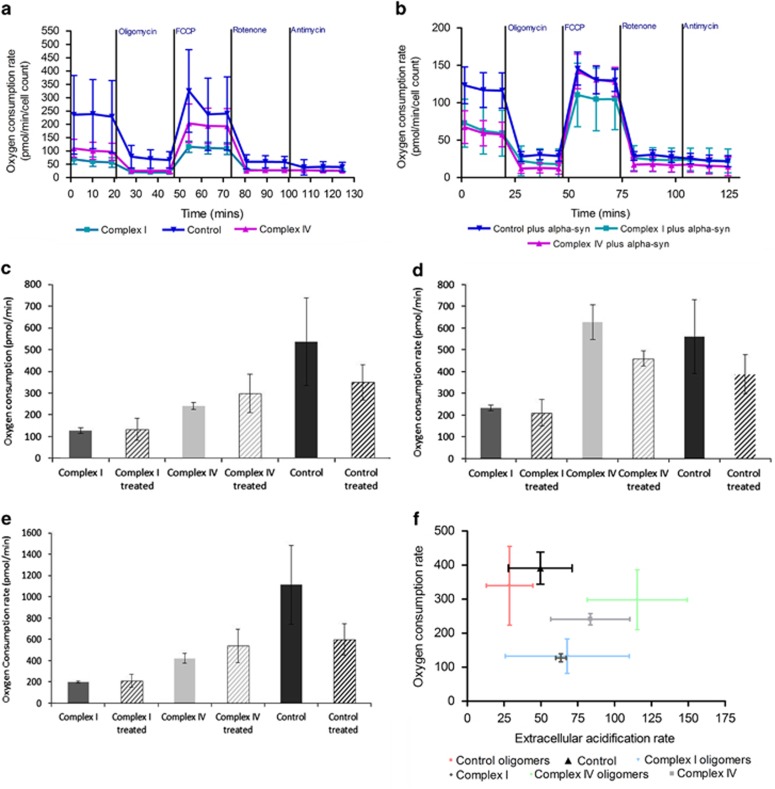
*α*-Synuclein affects the cellular respiration of neurons. Using a Seahorse extracellular flux analyser we examined the cellular respiration of neurons in the presence of aggregated *α*-synuclein. Oxygen consumption rates were measured at regular intervals with the addition of compounds to test mitochondrial activity, in (**a**) the absence or (**b**) presence of a 15-min pre-incubation with 40 nM aggregated *α*-synuclein. (**c**) Basal respiration is decreased in control neurons following treatment with aggregated *α*-synuclein. (**d**) Maximal respiration is also decreased following treatment in control and complex IV-deficient neurons. (**e**) ATP production through oxidative phosphorylation is also decreased in control cells upon treatment while it increases in complex IV neurons. Neurons harbouring a complex I defect show no changes in cellular respiration following treatment with aggregated *α*-synuclein. (**f**) Treatment with aggregated *α*-synuclein increases glycolysis in complex IV-deficient neurons, whereas have little effect on complex I-deficient neurons. Three replicates per cell line were performed

Using the Seahorse analyser (XFe24) we estimated the effect of aggregated *α*-synuclein on the respiration of neurons. The basal rate of respiration in neurons with complex I or complex IV deficiency was lower than in control neurons (Figures 3a and b) in both untreated cells and following 15 min pre-incubation of the neurons with aggregated *α*-synuclein (40 nM; three replicates per cell line). *α*-Synuclein had no effect on the basal rate of respiration of neurons with complex I or complex IV deficiency but reduced FCCP-induced maximal respiration in complex IV-deficient neurons (Figures 3c and d). Aggregated *α*-synuclein did not induce uncoupling in complex I- or complex IV-deficient cells (Figure 3e) but reduced the ratio of oligomycin-dependent and basal respiration that is more likely due to inhibition of the basal rate of respiration and not to the protonophoretic activity of the peptide (because it is absent in mutated cells). In addition, we can show that treatment with aggregated *α*-synuclein causes complex IV-deficient cells to become more glycolytic (Figure 3f), whereas decreasing the aerobic respiration of control cell lines and importantly showing very little effect on the complex I-deficient neurons.

### Aggregated *α*-synuclein affects the level of ATP in neurons

**Figure 4:**
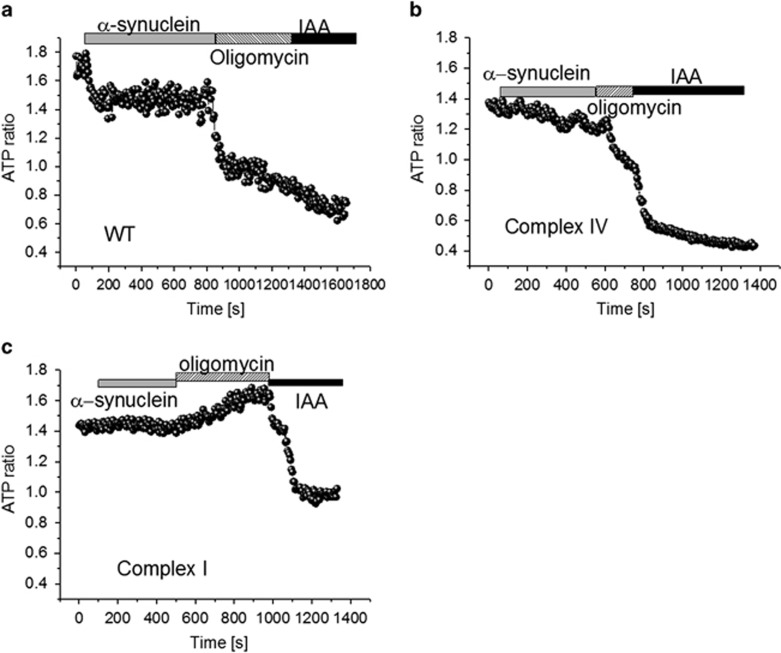
*α*-Synuclein affects the ATP production within neurons. Cytosolic ATP concentration in (**a**) control, (**b**) complex IV and (**c**) complex I-deficient neurons transfected with AT1.03. ATP levels are measured as the level of fluorescence and the ratio of the 527/475 nm signal in neurons after inhibition of oxidative phosphorylation by oligomycin (2 *μ*g/ml) and glycolysis by iodoacetic acid (IAA—20 *μ*M) following treatment of the cells with 100 nM *α*-synuclein. Basal levels of ATP are lower in cells with an inherent mitochondrial deficiency compared with control neurons. Treatment of neurons with aggregated *α*-synuclein causes a reduction in ATP levels in control neurons and those with a complex IV deficiency, but not in complex I-deficient neurons

The ability of *α*-synuclein to inhibit mitochondrial respiration may induce changes in [ATP]. We measured ATP levels inside neurons using a genetically encoded fluorescent ATP indicator (AT1.03),^19^ which we have previously and successfully used in primary neurons.^20^ We found that the basal level of ATP is lower in complex I-deficient neurons compared with control cells (1.43±0.11 ATP ratio; *n*=9, compared with 1.78±0.12 in controls; *P*<0.001). [ATP] in neurons with a complex IV defect was also decreased compared with controls (1.41±0.14 ratio compared with 1.78±0.12, *n*=8; *P*<0.001; Figures 4a and c). Application of aggregated *α*-synuclein (100 nM) significantly reduced the ATP level within control neurons (by 20%) or complex IV-deficient neurons (by 8%) but not in complex I-deficient neurons (Figures 4a and c). Subsequent application of inhibitors of oxidative phosphorylation and glycolysis (2 *μ*g/ml oligomycin and 20 *μ*M iodoacetic acid) blocked ATP production and induced a reduction in the ATP level of control neurons. Importantly, oligomycin induced an almost complete decrease of neuronal ATP in control cells suggesting that these cells produce ATP predominantly by oxidative phosphorylation. The effect of oligomycin was significantly smaller in complex IV-deficient neurons with a higher reliance on glycolysis. In complex I-deficient neurons, the effect of oligomycin was directly opposite to that on control cells—it increased the ATP level, suggesting that complex V is working in reverse mode in these cells and is therefore, consuming ATP for the maintenance of Δ*ψ*_m_. This finding confirms our previous observation of the effect of oligomycin on mitochondrial membrane potential.^16^ Thus, the effect of *α*-synuclein on the ATP level in cells with complex I mutation is less likely to be an initial trigger for neuronal cell death.

### The effect of aggregated *α*-synuclein on ROS production

**Figure 5:**
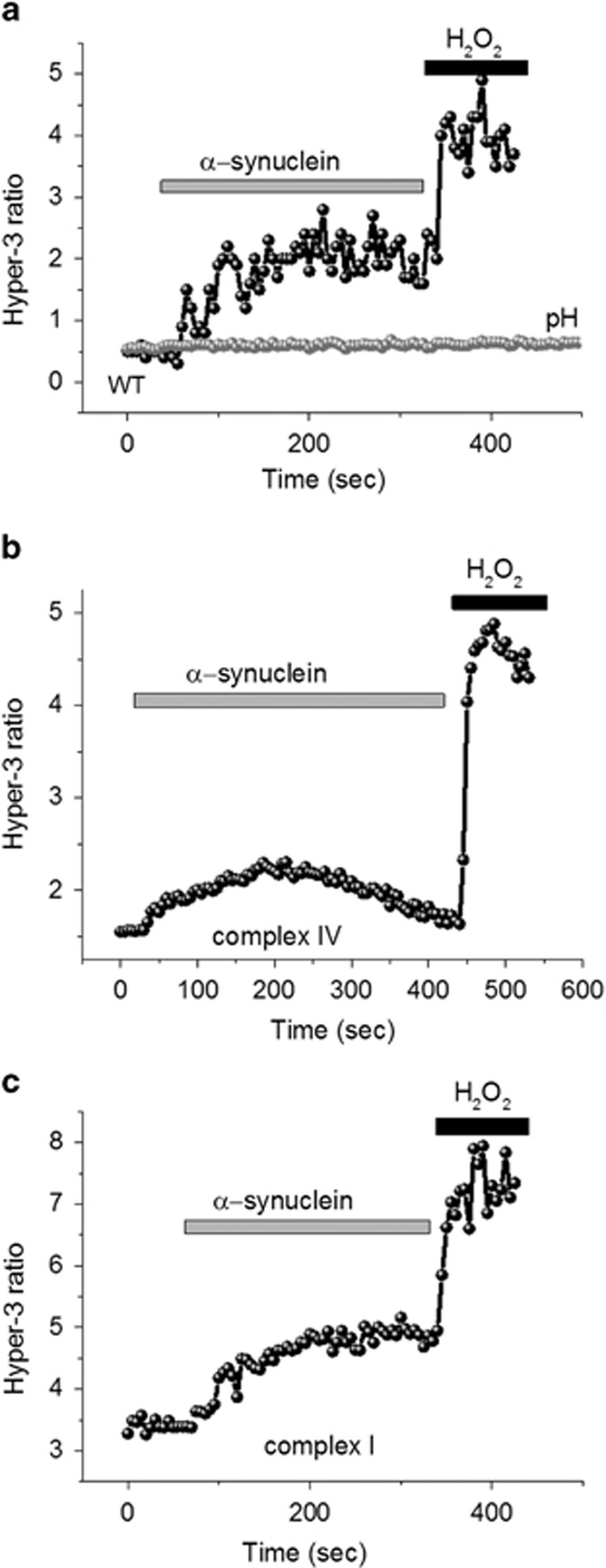
*α*-Synuclein affects ROS production within neurons. The level of intracellular hydrogen peroxide after application of 100 nM *α*-synuclein to (**a**) control neurons, (**b**) complex IV- or (**c**) complex I-deficient neurons. The grey trace in **a** represents Hyper- C199S ratio under application of *α*-synuclein and hydrogen peroxide. The basal level of ROS production was higher in neurons with a complex I or IV deficiency compared with control cells. Treatment with aggregated *α*-synuclein caused an increase in ROS production in control neurons and those with a complex I deficiency

Previously, we have shown that mitochondrial ROS is a main trigger for neuronal loss in cells with a complex I defect,^16^ and that oligomeric *α*-synuclein can induce cell death through induction of lipid peroxidation,^21^ therefore, we assessed the rate of hydrogen peroxide (H_2_O_2_) production, using the Hyper-3 genetic quantitative probe.^22^ In agreement with our previous publication, we observed that the basal level of H_2_O_2_ was significantly higher in complex I-deficient neurons (3.4±0.26 Hyper-3 ratio, *n*=4 experiments compared with 0.58±0.04, *n*=4; in control; *P*<0.001; Figures 5a and b). Hydrogen peroxide production in complex IV-deficient neurons was also increased compared with control neurons (1.4±0.11 Hyper-3 ratio; *n*=4; *P*<0.05; Figure 5c). Aggregated *α*-synuclein (100 nM) induced a similar increase in the H_2_O_2_ production of control neurons and cells with a complex I deficiency (Figures 5a and c). Note that effect of *α*-synuclein was not due to pH changes since the ROS independent but pH-dependent construct shows no changes in response to synuclein or hydrogen peroxide treatment (Figure 5a, grey line). Thus, *α*-synuclein induces similar changes in ROS production in all cell groups.

### *α*-Synuclein does not cause increased cell death in cells with a complex I deficiency

**Figure 6:**
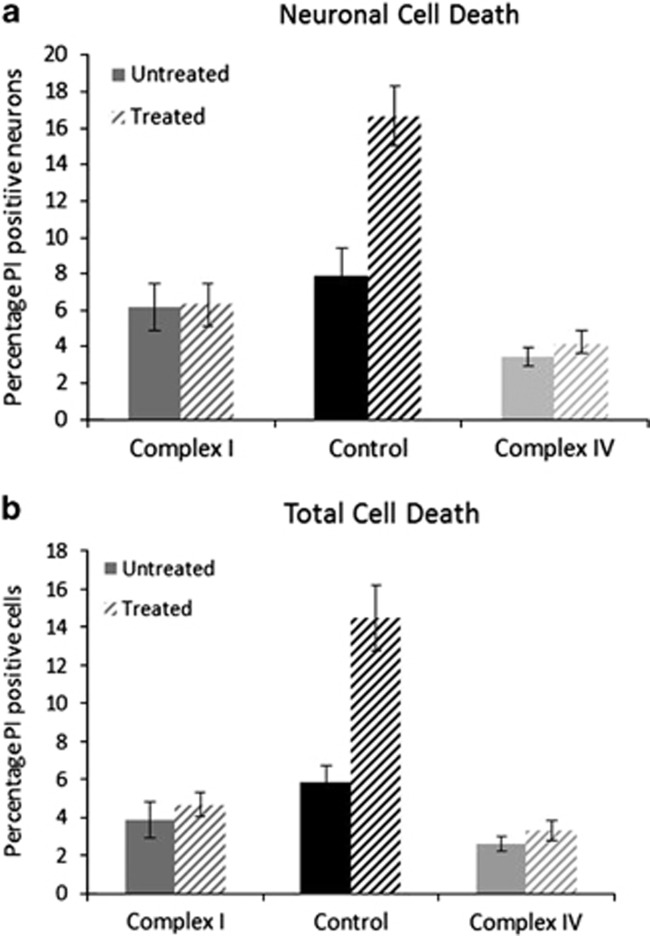
*α*-Synuclein does not affect the survival of neurons with mitochondrial defects. Using the ratio of Hoechst to propidium iodide staining, we estimated cell death following treatment with aggregated *α*-synuclein in both (**a**) neurons and (**b**) the total cell population. Treatment with 500 nM *α*-synuclein for 24 h was associated with increased cell death in control cells but not in cells with an inherent mitochondrial defect. **P=0.0016, ***P=0.0001

To measure the effects of *α*-synuclein on cell survival, we treated undifferentiated and neuronal cells with 500 nM aggregated *α*-synuclein for 24 h, following this, cells were loaded with Hoechst, to give total cell counts, and propidium iodide (PI), to give counts of dead cells. Using this approach we calculated the percentage cell death that had occurred following treatment and because of the uptake of PI we could measure those cells that had died via necrosis (Figure 6). Using brightfield images we could also compare the amount of neuronal cell death that had occurred *versus* the death of other cell types within the culture. This experiment revealed that aggregated *α*-synuclein treatment drastically increases the amount of cell death for control neurons (*n*=5 images, *n*=~4300 cells), with cell death doubling from 8 to 16% (*P*=0.0016) following 24 h incubation with aggregated *α*-synuclein (Figure 6a). However, incubation with aggregated *α*-synuclein did not cause an increase in neuronal cell death of complex I- or complex IV-deficient cell lines (Figure 6a; *n*=5 images, *n*=~2500 (complex I, *P*=0.992) and ~7200 (complex IV, *P*=0.457)). Furthermore, we can look at total cell death including undifferentiated cells within the same culture and a similar observation was made, 24 h treatment with aggregated *α*-synuclein cause a significant increase in cell death for control cells (*n*=5 images, *n*=1700) with the percentage cell death rising from 5.8 to 14.5% (*P*=0.0001). Consistent with the neuronal data there was no increase in total cell death recorded for complex I- (*P*=0.54) or complex IV- (*P*=0.27) deficient cells. These data are consistent with other experimental data, which suggest that since oligomers inhibit complex I they are not detrimental to cells already harbouring a complex I defect. These data suggest that *α*-synuclein and mitochondrial defects (due to mitochondrial DNA mutations) within the same cell do not cause necrotic cell death.

## Discussion

Both *α*-synuclein and mitochondrial dysfunction have been proposed to be important for the loss of dopaminergic, SN neurons in PD and studies have suggested that inhibition of mitochondrial complex I is fundamental to this loss. We aimed to further understand the relationship between mitochondria, aggregated *α*-synuclein and cell death by studying the effects of aggregated *α*-synuclein on neurons with deficiencies in mitochondrial complex I or IV. The *α*-synuclein used in these experiments is based on an aggregation procedure that generates a mixture of monomers and oligomeric forms, as previously described,^17^ in a 99% to 1% ratio. The oligomeric species have been characterised to be compact *β*-sheet structure oligomers. We utilised this mixed species preparation as it most accurately reflects the endogenous *α*-synuclein within the intracellular environment in PD, that is, a mixture of species that are predominantly monomeric with a small proportion of presumed highly toxic *β-*sheet oligomers. Furthermore, we are able to determine the specific effect of the oligomers within the preparation by performing monomer only experiments. These data show that aggregated forms of *α*-synuclein inhibit complex I of the electron transport chain, have little effect on cells with inherent complex I deficiency and importantly do not cause cell death in these neurons.

### Aggregated *α*-synuclein impairs mitochondrial function through complex I inhibition

*α*-Synuclein is capable of inducing mitochondrial defects in several models where mutant *α*-synuclein is overexpressed, for example, Martin *et al* have shown that overexpression of *α*-synuclein reduces activity of mitochondrial complex IV.^23^ Although mutant *α*-synuclein is capable of binding to voltage-dependent anion channels and interacting with the mitochondrial adenylate translocator to facilitate mitochondrial permeability transition pore opening and modulation of the PD phenotype in experimental mice.^24, 25^ The dysfunction induced in isolated mitochondria by oligomeric forms of *α*-synuclein is both complex I-dependent and calcium-mediated,^13^ whereas mice that overexpress *α*-synuclein show age dependent, differential defects of mitochondrial respiration in different brain areas.^26^ These effects on mitochondrial complex I have been shown to be ameliorated by overexpression of TOM20.^27^ Mutated forms of *α*-synuclein cause mitochondrial structural changes, particularly affecting the cristae,^28^ whereas *Drosophila* models have highlighted the contribution of oxidative stress to the neuronal changes seen, for example, the expression of human *α*-synuclein bearing the A53T or A30P mutations leads to hypersensitivity to hypoxia.^29^ Although transgenic mice expressing mutant forms of *α-*synuclein have also been generated, they often do not show SN dopaminergic neuronal loss despite very severe phenotypes and neuron loss in other brain regions.^23^

Here we investigate the interaction of *α*-synuclein with mitochondria in more detail and show that aggregated *α*-synuclein reduces mitochondrial membrane potential, inhibits mitochondrial respiration and ATP production in control cells, but not in those with a complex I defect. The complex I cybrids used in this study have previously been shown to have adapted to their deficiency by upregulating mitochondrial membrane potential through the reversal of complex V.^16^ This means that further inhibition via aggregated *α*-synuclein has little impact on the function or survival of these cells. Previous studies using these cells have also shown that the level of ROS production within these cybrids is higher than in control cell lines.^16^ We show here that aggregated *α*-synuclein further increases the production of ROS within these cell lines, but again that there is no enhancement of the effect in complex I-deficient cells.

### Acute complex I inhibition by *α*-synuclein causes cell loss, but not in neurons with inherent, chronic complex I deficiency

These data are consistent with previous studies that investigated the effect of *α*-synuclein on human dopaminergic neurons.^9^ The import of *α*-synuclein into mitochondria is an energy-dependent process requiring an intact membrane potential,^9^ thus despite the respiratory defects within the complex I-deficient cells import of *α*-synuclein is still possible due to the increase in membrane potential of these cells. Importantly, we show that the combination of mitochondrial dysfunction and inherent complex I deficiency is not sufficient to cause neuronal loss. Acute complex I inhibition, such as occurs following *α*-synuclein treatment, does cause neuronal loss, but long-term chronic complex I inhibition, such as may occur during ageing, is not associated with cell death in the presence of *α*-synuclein.

A reduction in complex I activity and expression has been reported in the SN of patient's with PD and inhibitors of this complex (such as rotenone and mitochondrial permeability transition pore) induce a parkinsonian phenotype in animal models,^30, 31, 32^ change the expression of *α*-synuclein^33^ and effect the aggregation of *α*-synuclein even in the absence of mitochondrial complex I.^34^ Therefore, the inhibition of complex I by aggregating *α*-synuclein could be a key step in the pathogenesis of PD. Our data suggest that although complex I defects are detrimental to cells, the accumulation of *α*-synuclein does not enhance any defects when differentiated neurons have an existing inhibition of complex I. In culture, these cells have adapted their physiology to be able to survive, predominantly through the reversal of the ATP synthase (complex V), therefore further inhibition of complex I by aggregated *α*-synuclein does not have a toxic effect. Complex I inhibition by *α*-synuclein is more marked in control cells and those with a complex IV defect as these neurons have not encountered such inhibition before and are therefore more sensitive.

## Conclusions

Aggregated *α*-synuclein inhibits mitochondrial complex I leading to reduced respiration and ATP production in neurons alongside an increase in ROS production. A pre-existing deficiency in complex I does not enhance these defects.

## Materials and Methods

### *α*-Synuclein

All forms of *α*-synuclein used in this study were generated by our collaborators, the manufacture and purification of this protein are detailed in Cremades *et al.*^17^

### Cell culture

The cell lines used in this study have been previously described.^35^ All cybrid lines were derived from the parental line CC9.3.1, used as the control for this study. Cybrids were generated with a severe complex I deficiency due to two homoplasmic mtDNA mutations in complex I (m.13887Cins in MTND6 and m.12273G>A in MTND5) and with a mild complex IV deficiency due to a homoplasmic mtDNA mutation in MTCO1 (m.6589T>C). The cells were passaged and differentiated as previously described ^35^ and using the 4+/4− method.^36^

### Measurement of mitochondrial membrane potential

Mitochondrial membrane potential (Δ*ψ*_m_) measurements were made for untreated cells as a baseline and those treated with aggregated forms of *α*-synuclein as described previously.^16^ Briefly, measurements were made on cells loaded with 25 nM tetramethylrhodamine methyl ester (TMRM, Life Technologies, Pisley, Scotland, UK) for 40 min before imaging in Hank's balanced salt solution (Life Technologies), 25 nM TMRM was maintained for imaging. TMRM fluorescence intensity was quantified by measuring the peak TMRM fluorescence intensity for single mitochondria within a given cell. For each cell line and treatment, a minimum of three images were taken and within each image a minimum of six measurements of distinct mitochondrial regions were taken to give *n*=18.

### Measurement of cellular respiration

Mitochondrial respiratory activity was measured in live neurons in a Seahorse XFe24 extracellular flux analyser (Seahorse Bioscience, North Billerica, MA, USA). Neurons were seeded onto a 24-well plate (50 000 cells/well) 24 h before the experiment. Growth media were replaced with basic experimental media (plus glucose) supplemented with FBS to 2% and 10 mM pyruvate (pH 7.4) 1 h before the experiment and the neurons incubated in a CO_2_-free atmosphere. Measurements of O_2_ consumption rate (OCR) and extracellular acidification rate were taken, mitochondrial activity was tested using: oligomycin (to 1.3 *μ*M, an ATP synthase inhibitor), carbonyl cyanide p-trifluoromethoxy-phenylhydrazone (FCCP, a respiratory chain uncoupler to drive maximum respiratory capacity (5 *μ*M), rotenone (1 *μ*M, a complex I inhibitor) and finally antimycin A (to 1 *μ*M), which blocks complex III.^37^ Data were normalised by cell number, and a number of parameters were assessed, including basal respiration, maximal respiration following FCCP treatment and ATP production by oxidative phosphorylation which was calculated by multiplying ATP turnover ((basal OCR—NMR)—(oligomycin-inhibited OCR – NMR)) by the established phosphorus/oxygen (P/O) ratio of 2.3.^38^ A total of three replicates per cell line were performed.

### Measurement of NADH /redox indexes

NADH autofluorescence was measured using a Zeiss 710 VIS CLSM (Carl Zeiss Microscopy, Munich, Germany) equipped with a META detection system and a x40 oil immersion objective. Membrane potential, ROS production, intracellular ATP and cell death measurements were all taken using this microscopy setup. For NADH autofluorescence, excitation light of 405 nm and emitted fluorescence measured at 420–450 nm. Illumination intensity was kept to a minimum (at 0.1–0.2% of laser output) to avoid phototoxicity and the pinhole set to give an optical slice of ~2 *μ*m. The addition of FCCP (final concentration 2 μM) gave maximal respiration, whereas 1 mM cyanide elicited minimal respiration. Data were then normalised to data from untreated control cells and the NADH mitochondrial pool and percentage redox indexes calculated.

### Measurement of ROS

For intracellular H_2_O_2_ assessments, cells were transfected with the Hyper-3 construct or the Hyper-CS control pH probe using Effectene according to the manufacturers' instructions (Qiagen, Manchester, UK). Importantly, due to the toxicity of the Effectene reagent in combination with Neurobasal A media (Life Technologies), cells were exposed to the transfection complexes for an hour before the media were replaced with normal Neurobasal A growth medium. Cells were then left to express the constructs for 48 h before experiments were performed. Hyper-3 or Hyper-C199S fluorescence was excited at 488 and 405 nm and emission set at 510–540 nm and expressed as 488/405 ratio.

### Imaging of intracellular ATP levels with FRET-based genetically encoded indicator AT1.03

Cells were transfected with AT1.03 and mitAT1.03 plasmids (Addgene, Cambridge, MA, USA) as described above. Measurements of ATP levels with AT1.03 and mitAT1.03 were performed on a confocal microscope and images were obtained using a x63 oil immersion objective to allow measuring the fluorescent signal immediately over dendrites. Cyan fluorescent protein was excited with 405 nm, and emission from 460 to 510 nm was scanned. The 405-nm laser line was used to excite yellow fluorescent protein, which was measured using a bandpass filter from 515 to 580 nm. Illumination intensity was kept to a minimum (at 0.1–0.2% of laser output) to avoid phototoxicity and the pinhole set to give an optical slice of ∼2 *μ*m.

### Cell death

Following 24 h of treatment with *α*-synuclein cell death was estimated using Hoechst (Life Technologies) and propidium iodide (Life Technologies). The cells were incubated with 2 *μ*M propidium iodide and 5*μ*M Hoechst in Hank's balanced salt solution for 30 min. Single images were then captured, five per cell line, the total number of dead cells (propidium iodide positive) counted and the percentage cell death calculated as a percentage of the total cells (Hoechst positive).

### Statistical analysis

Data sets were tested for normality using the Kolmogorov–Smirnov, D'Agostino and Pearson omnibus and Shapiro–Wilk tests. Normal data sets were then analysed for statistical significance using a paired *t*-test and non-normal sets using a Mann–Whitney *U*-test.

## Abbreviations

FCCP: carbonyl cyanide p-trifluoromethoxy-phenylhydrazone
FRET: Fluorescence resonance energy transfer
mtDNA: mitochondrial DNA
NADH: nicotinamide adenine nucleotide
OCR: O_2_ consumption rate
PD: Parkinson's disease
ROS: reactive oxygen species
SN: substantia nigra
TMRM: tetramethylrhodamine methyl ester
